# Two decades of family planning in Ethiopia and the way forward to sustain hard-fought gains!

**DOI:** 10.1186/s12978-022-01435-5

**Published:** 2022-06-13

**Authors:** Mengistu Asnake Kibret, Lia Tadesse Gebremedhin

**Affiliations:** 1 Pathfinder International, Addis Ababa, Ethiopia; 2 Federal Ministry of Health, Addis Ababa, Ethiopia

## Abstract

Family planning (FP) is a human right, and ensuring women’s access to FP is central to protecting the health and wellbeing of mothers and children. Over the past two decades, Ethiopia has made FP service more widely available, increasing the contraceptive prevalence rate from 8% in 2000 to 41% in 2019. This remarkable fivefold increase can be attributed to the country’s overall development, including investment in education (particularly for girls) and reduction in child marriage, as well as the adoption and implementation of several enabling FP policies and strategies. In Ethiopia, achieving universal access to sexual and reproductive health care services, information, and education, including FP, by 2030 means enhancing these effective government policies and programs. Achieving universal access requires increasing financial resources, including domestic financing through greater government commitment for commodity security and program implementation; strengthening public–private partnerships; and improving service delivery for populations that are hard to reach and/or in humanitarian crisis. The persistence of equity gaps due to regional and/or sociodemographic disparities and the low quality of FP service delivery challenge our progress in Ethiopia. The papers included in this supplement provide additional detail on the overall progress described in this commentary and highlight focal areas for improvement in responding to unmet needs. Current policies and services must adapt, maintain, and build upon these gains and focus on targeted actions in areas identified for improvement. We must sustain the hard-fought gains of the past decades and help shape the prosperous future we advocate for in our society by 2030 and beyond—Leaving No One Behind.

## Introduction

Family planning (FP) is a human right, and ensuring women’s FP access is central to protecting the health and wellbeing of mothers and children. The Constitution of the Federal Democratic Republic of Ethiopia Article 35(9) clearly sets forth a woman’s right to FP, stating that “[t]o prevent harm arising from pregnancy and childbirth and in order to safeguard their health, women have the right of access to family planning education, information and capacity” [1].

Although the 1994 constitution set forth a right to family planning, prior to 2000, the FP program in Ethiopia suffered from a shortage of trained personnel, limited contraceptive commodity supplies, and inadequate supervisory support and monitoring systems: all impacted access to and quality of service provision, evidenced by a low contraceptive prevalence rate [2]. However, over the past two decades, Ethiopia has made the remarkable achievement of increasing the contraceptive prevalence rate from 8% in 2000 [3] to 41% in 2019 [4]. This fivefold increase can be attributed to the country’s overall development, including investment in education (particularly for girls) and reduction in child marriage, as well as the adoption and implementation of several enabling FP policies and strategies. Policies that support FP services were anchored largely on the principles of the 1987 Safe Motherhood Initiative [5] and the Millennium Development Goals (MDGs) [6]. More recently, Ethiopia’s FP2020 commitment, made at the 2012 London Summit on Family Planning, further shaped the national FP strategies [7].

Starting in the early 1990s, the government of Ethiopia worked to shape an enabling environment for prioritizing FP services through various sectoral reforms [8–14]. In the late 1990s and early 2000s, the government implemented these different sectoral reforms and specific policies by developing different strategic documents. Leaders established a cornerstone policy, the Ethiopian flagship health extension program (HEP) [15, 16]. HEP built on the experience of community-based FP programs implemented by international and local nongovernmental organizations (NGOs) in the late 1990s. Among them, the Family Guidance Association of Ethiopia and Consortium of Reproductive Health Associations played major roles in providing services and advocating for better policies and strategies [2]. These NGO-led programs laid the foundation to expanding women’s and girls’ access to FP information, counseling, and services in Ethiopia. However, the high dropout rates of community volunteers, lack of established incentive mechanisms, and resource scarcity led NGOs to phase out many community-based FP programs [17–19]. The programs’ experience, however, informed HEP, which has been instrumental in accelerating progress to meet the country’s MDGs [20]. HEP was also critical to increasing the uptake of long-acting reversible contraceptives [21]. Innovative service delivery approaches that include social marketing and using a network of private sector outlets also contribute to increasing access and responding to unmet needs [22, 23].

The Ethiopian government’s decisions in 2007 to remove the tax levied on contraceptives [24] and empower the Ethiopian Pharmaceutical Supply Agency to procure and distribute contraceptives facilitated contraceptive supply and access [25]. At the same time, the government also broadened the resource base by increasing domestic financial resources allocated to the FP program: the annual allocation to the FP budget has continued to grow over the last decade. The country’s FP program, however, still largely depends on external resources [26]. Advocates must push for continued increase of government financial commitments to FP and reproductive health programs.

The United Nations agenda on Leaving No One Behind is adopted as the vision, promise, and commitment of the Ethiopian government’s 2030 Agenda and the SDGs [27, 28]. In Ethiopia, achieving universal access to sexual and reproductive health care services, information, and education, including FP, by 2030 means enhancing these effective government policies and programs. Achieving universal access requires increasing financial resources, including domestic financing through greater government commitment for commodity security and program implementation; strengthening public–private partnerships; and improving service delivery for populations that are hard to reach and/or in humanitarian crisis [29].

Two challenges block further FP progress in Ethiopia: the persistence of equity gaps due to regional and/or sociodemographic disparities and the low quality of FP service delivery. Government agencies should fine tune their growth and development policies to close these gaps and ensure access to quality services for the entire population, in particular youths. The political commitment to incorporate FP into the development arena, as demonstrated in the health sector transformation plan and Ethiopia’s other national development plans, will help address this unfinished agenda. In addition, improving the quality of FP service delivery, expanding effective models of service integration, and reaching hard-to-reach population groups or subgroups and people in humanitarian crisis will further enhance the success of FP programs.

The papers included in this supplement provide additional detail on the overall progress described in this commentary and highlight focal areas for improvement in responding to unmet needs. These areas include child marriage in hot-spot areas, gender inequality, adolescent reproductive health, improving quality of care, and counteracting widespread individual and community misperceptions and beliefs. The emergence of the COVID-19 pandemic and other human-produced and natural disasters in the past years might affect the advancement of the overall achievements gained so far. We must adapt our work to maintain these gains. In addition, targeted actions in areas identified for improvement will sustain the hard-fought gains in the past decades and help shape the prosperous future we advocate for in our society by 2030 and beyond—Leaving No One Behind.

## Data Availability

Data sharing is not applicable to this article because no data sets were generated or analyzed during the current study.

## Abbreviations

FP: Family planning
HEP: Health extension program
MDG: Millennium development goal
NGO: Nongovernmental Organization
SDG: Sustainable development goals

## References

[1] Federal Democratic Republic of Ethiopia. Constitution of the Federal Democratic Republic of Ethiopia Proclamation No. 1/1995. Federal Negarit Gazeta, 21st day of August 1995; Addis Ababa, Ethiopia.

[2] Antenane K (1998). Situational analysis of family planning services in Ethiopia. Ethiopian J Health Dev.

[3] CSA [Ethiopia] and ORC Macro: Ethiopia Demographic and Health Survey 2000. 2001, Addis Ababa, Ethiopia and Calverton, Maryland, USA: Central statistical Agency and ICF International; Available at http://dhsprogram.com/pubs/pdf/FR118/FR118.pdf. Accessed 20 Apr 2020.

[4] Public Health Institute, Ministry of Health and ICF. Ethiopia Mini Demographic and Health Survey 2019. 2021; Addis Ababa, Ethiopia: EPHI/FMoH/ICF; Available at https://www.dhsprogram.com/pubs/pdf/FR363/FR363.pdf. Accessed 20 Apr 2020.

[5] Mahler H (1987). The safe motherhood initiative: a call to action. Lancet.

[6] United nation. Resolution adopted by the general assembly, 55/2. United Nations Millennium Declaration. Retrieved June 2000; 4:2015.

[7] Brown W, Druce N, Bunting J, Radloff S, Koroma D, Gupta S (2014). Developing the "120 by 20" goal for the Global FP2020 Initiative. Stud Fam Plann.

[8] Ministry of Health [Ethiopia]. Health Policy of the Transitional Government of Ethiopia. 1993; Addis Ababa, Ethiopia; Available at http://repository.iifphc.org/handle/123456789/210. Accessed 20 Apr 2020.

[9] Ministry of Education [Ethiopia]. Education and Training Policy of the Transitional Government of Ethiopia EMPDA. 1994; Addis Ababa, Ethiopia.

[10] Federal Democratic Republic of Ethiopia. National Population Policy of Ethiopia. 1993; Addis Ababa: Office of the Prime Minister, FDRE.

[11] Federal Democratic Republic of Ethiopia. Women’s Policy of the Transitional Government of Ethiopia. 1993; Addis Ababa; Ethiopia: FDRE.

[12] Ministry of Youth, Sports and Culture [Ethiopia]. National Youth Policy. 2004; Addis Ababa: MOYSC.

[13] Ministry of Finance and Economic Development [Ethiopia]. Building on Progress a Plan for Accelerated and Sustained Development to end Poverty (PASDEP) 2005/06–2009/10.2005b; Addis Ababa, Ethiopia MOFED.

[14] Ministry of Health [Ethiopia]. Health Sector Transformation Plan (2015/16 - 2019/20). 2016; Addis Ababa, Ethiopia.

[15] Ministry of Health [Ethiopia]. Health Sector Development Program-HSDP II: 2002/03–2004/05. Report of the final evaluation of the HSDP II. 2006; Addis Ababa, Ethiopia.

[16] Ministry of Health [Ethiopia]. Health Extension Program Implementation Guideline, Revised. 2004; Addis Ababa, Federal Ministry of Health in Ethiopia.

[17] Tawye Y, Jotie F, Shigu T, Ngom P, Maggwa N (2005). The potential impact of community-based distribution programmes on contraceptive uptake in resource-poor settings: evidence from Ethiopia. Afr J Reprod Health.

[18] Mitike G (2000). Community-based distribution of family planning as perceived by people in the reproductive age group, North and South Gondar Zones, Ethiopia. Ethiopian J Health Dev.

[19] Argaw D, Fanthahun M, Berhane Y (2007). Sustainability and factors affecting the success of community-based reproductive health programs in rural northwest Ethiopia. Afr J Reprod Health.

[20] Assefa Y, Gelaw YA, Hill PS, Taye BW, Van Damme W (2019). Community health extension program of Ethiopia, 2003–2018: successes and challenges toward universal coverage for primary healthcare services. Global Health.

[21] Asnake M, Henry EG, Tilahun Y, Oliveras E (2013). Addressing unmet need for long-acting family planning in Ethiopia: uptake of single-rod progestogen contraceptive implants (Implanon) and characteristics of users. Int J Gynaecol Obstet.

[22] Ahmed J, Genet M. Evaluation of Program Options to Meet Unmet Need for Family Planning in Ethiopia. 2002; Calverton, Maryland USA: ORC Macro.

[23] Riley C, Garfinkel D, Thanel K, Esch K, Workalemahu E, Anyanti J, et al. Getting to FP2020: Harnessing the private sector to increase modern contraceptive access and choice in Ethiopia, Nigeria, and DRC. PLoS One. 2018;13(2). 10.1371/journal.pone.0192522. 10.1371/journal.pone.0192522 PMC5812628 29444140

[24] Olson DJ, Piller A (2013). Ethiopia: an emerging family planning success story. Stud Fam Plann.

[25] Federal Democratic Republic of Ethiopia. Drug Fund and Pharmaceutical Supply Agency Proclamation: Federal Negarit Gazeta Proclamation No. 553/2007. 2007; Addis Ababa, Ethiopia. Available: https://chilot.me/wp-content/uploads/2011/01/proc-no-553-drug-fund-and.pdf. Accessed 20 Apr 2020.

[26] Ministry of Health [Ethiopia]. Ethiopia Health Accounts 2016/17. 2019, Addis Ababa, Ethiopia.

[27] United Nations World Fertility and Family Planning 2020: Highlights (ST/ESA/SER.A/440). 2020, population division, Department of Economic and Social Affairs.

[28] Sachs J, Schmidt-Traub G, Kroll C, Lafortune G, Fuller G. Sustainable Development Report. 2019; New York: Bertelsmann Stiftung and Sustainable Development Solutions Network (SDSN).

[29] National Planning Commission [Ethiopia]. Ethiopia 2017 Voluntary National Review on SDGs: Government Commitments, National Ownership and Performance Trends. Addis Ababa: Federal Democratic Republic of Ethiopia National Planning Commission. 2017:1–45.

